# Estimating the Global Burden of Endemic Canine Rabies

**DOI:** 10.1371/journal.pntd.0003709

**Published:** 2015-04-16

**Authors:** Katie Hampson, Laurent Coudeville, Tiziana Lembo, Maganga Sambo, Alexia Kieffer, Michaël Attlan, Jacques Barrat, Jesse D. Blanton, Deborah J. Briggs, Sarah Cleaveland, Peter Costa, Conrad M. Freuling, Elly Hiby, Lea Knopf, Fernando Leanes, François-Xavier Meslin, Artem Metlin, Mary Elizabeth Miranda, Thomas Müller, Louis H. Nel, Sergio Recuenco, Charles E. Rupprecht, Carolin Schumacher, Louise Taylor, Marco Antonio Natal Vigilato, Jakob Zinsstag, Jonathan Dushoff, on behalf of the Global Alliance for Rabies Control Partners for Rabies Prevention

**Affiliations:** 1 Boyd Orr Centre for Population and Ecosystem Health, Institute for Biodiversity, Animal Health & Comparative Medicine, University of Glasgow, Glasgow, United Kingdom; 2 Sanofi Pasteur, Lyon, France; 3 Ifakara Health Institute, Ifakara, Tanzania; 4 ANSES—French Agency for Food, Environmental and Occupational Health and Safety, Rabies and Wildlife laboratory of Nancy, Atton, France; 5 Centers for Disease Control and Prevention, Atlanta, Georgia, United States of America; 6 Global Alliance for Rabies Control, Manhattan, Kansas, United States of America; 7 Friedrich-Loeffler-Institute—Federal Research Institute for Animal Health, Greifswald—Insel Riems, Germany; 8 International Companion Animal Management Coalition, Cambridge, United Kingdom; 9 Pan-American Health Organization, Rio de Janeiro, Brazil; 10 Independent rabies scientist, Geneva, Switzerland; 11 Institut Pasteur in Cambodia, Phnom Penh, Cambodia; 12 University of Pretoria, Pretoria, South Africa; 13 Ross University School of Veterinary Medicine, Basseterre, St. Kitts, West Indes; 14 Merial, Lyon, France; 15 Swiss Tropical and Public Health Institute, Basel, Switzerland; 16 McMaster University, Hamilton, Ontario, Canada; Oswaldo Cruz Foundation, BRAZIL

## Abstract

**Background:**

Rabies is a notoriously underreported and neglected disease of low-income countries. This study aims to estimate the public health and economic burden of rabies circulating in domestic dog populations, globally and on a country-by-country basis, allowing an objective assessment of how much this preventable disease costs endemic countries.

**Methodology/Principal Findings:**

We established relationships between rabies mortality and rabies prevention and control measures, which we incorporated into a model framework. We used data derived from extensive literature searches and questionnaires on disease incidence, control interventions and preventative measures within this framework to estimate the disease burden. The burden of rabies impacts on public health sector budgets, local communities and livestock economies, with the highest risk of rabies in the poorest regions of the world. This study estimates that globally canine rabies causes approximately 59,000 (95% Confidence Intervals: 25-159,000) human deaths, over 3.7 million (95% CIs: 1.6-10.4 million) disability-adjusted life years (DALYs) and 8.6 billion USD (95% CIs: 2.9-21.5 billion) economic losses annually. The largest component of the economic burden is due to premature death (55%), followed by direct costs of post-exposure prophylaxis (PEP, 20%) and lost income whilst seeking PEP (15.5%), with only limited costs to the veterinary sector due to dog vaccination (1.5%), and additional costs to communities from livestock losses (6%).

**Conclusions/Significance:**

This study demonstrates that investment in dog vaccination, the single most effective way of reducing the disease burden, has been inadequate and that the availability and affordability of PEP needs improving. Collaborative investments by medical and veterinary sectors could dramatically reduce the current large, and unnecessary, burden of rabies on affected communities. Improved surveillance is needed to reduce uncertainty in burden estimates and to monitor the impacts of control efforts.

## Author Summary

Rabies is a fatal viral disease largely transmitted to humans from bites by infected animals—predominantly from domestic dogs. The disease is entirely preventable through prompt administration of post-exposure prophylaxis (PEP) to bite victims and can be controlled through mass vaccination of domestic dogs. Yet, rabies is still very prevalent in developing countries, affecting populations with limited access to health care. The disease is also grossly underreported in these areas because most victims die at home. This leads to insufficient prioritization of rabies prevention in public health agendas. To address this lack of information on the impacts of rabies, in this study, we compiled available data to provide a robust estimate of the health and economic implications of dog rabies globally. The most important impacts included: loss of human lives (approximately 59,000 annually) and productivity due to premature death from rabies, and costs of obtaining PEP once an exposure has occurred. The greatest risk of developing rabies fell upon the poorest regions of the world, where domestic dog vaccination is not widely implemented and access to PEP is most limited. A greater focus on mass dog vaccination could eliminate the disease at source, reducing the need for costly PEP and preventing the large and unnecessary burden of mortality on at-risk communities.

## Introduction

Rabies is a fatal viral infection that can infect all mammals, but domestic dogs cause over 99% of all human deaths from rabies [1]. Human rabies can be prevented through prompt administration of post-exposure prophylaxis (PEP) to victims of bites by rabid animals [2], and infection can be eliminated at source through sustained mass vaccination of reservoir populations [3]. Most industrialized countries have eliminated rabies from domestic dog populations. However, in the majority of developing countries, rabies remains endemic in domestic dog populations and poorly controlled [4]. Our focus is on the impacts of canine-adapted variants of the rabies virus, sustained predominantly or entirely by transmission in domestic dogs (it is unclear whether independent transmission in wildlife might be sufficient for maintenance in some areas [5–7]). Our definition therefore includes rabies cases or exposures caused by canine variants of rabies virus also transmitted from wildlife, though these are negligible compared to those transmitted by domestic dogs.

The human and economic costs of canine rabies are poorly known [1]. A major challenge to estimating the burden of rabies is the absence of reliable surveillance data for countries where the disease is most prevalent. Basic information on how many lives are lost to rabies and the economic costs of preventing disease amongst those exposed are needed to advocate for sustainable control programmes. Official reporting of incidence data on rabies and rabies exposures remains desperately poor in most canine rabies-endemic countries, and is increasingly recognized to grossly underestimate the true number of cases [8, 9]. Active surveillance studies highlight the disparities between officially recorded and likely occurring rabies deaths. These include recent studies from both Asia and Africa based on probability decision tree modelling [10, 11]; extensive verbal autopsy surveys [12]; community surveys [13, 14] and contact tracing [15], which all show much higher mortality than officially reported.

Specific features of rabies contribute to the problem of underreporting. Death is inevitable following clinical onset and therefore a large number of rabies victims never report to health facilities and are never diagnosed. Misdiagnosis to other neurological syndromes is frequent, especially in malaria endemic regions [16]. Shortages of life-saving PEP [15] and centres that provide PEP for bite victims [17] and poorly monitored sales of PEP to private suppliers all complicate counting the number of rabies diagnoses made and the number of treatments given. These problems of PEP provision particularly increase the risks of disease among the rural poor, an already marginalized sector of society. Moreover, poor infrastructure and a lack of personnel and facilities for rabies surveillance and diagnosis in most developing countries means that only very limited data of questionable reliability are available.

In the absence of either reliable mortality reporting systems or more widespread active surveillance studies, extrapolations are required to estimate the global burden of rabies. Predictive methods have been developed to overcome the underreporting of disease, including a probability decision-tree method to determine the likelihood of the onset of clinical rabies in humans following the bite of a suspect rabid dog [8]. Using this method and drawing on data from a limited number of countries, Knobel *et al*. estimated that canine rabies caused approximately 55,000 human deaths annually across Africa and Asia [18]. Since this 2005 study, more data have become available and the disease situation has changed, with concerted control efforts in some parts of the world [19], increased incidence in others [20], as well as emergence in previously rabies-free areas [21, 22]. An updated assessment of the global rabies burden is therefore required.

The rabies burden is made up of different components. Societal costs include mortality and lost productivity from premature death, and morbidity from adverse events (AE) of vaccination using nerve tissue vaccines (NTVs) and psychological effects of exposure to this fatal disease, expressed as disability-adjusted life years (DALYs). Direct costs of PEP (depending on the use of rabies immunoglobulin (RIG), and the type of vaccine and regimen, for example intramuscular (IM) versus intradermal (ID) administration) and indirect costs of seeking PEP (travel and accommodation for multiple clinic visits and lost income) fall upon the medical sector and affected communities, whilst the veterinary sector typically incurs costs related to dog vaccination. Veterinary and medical sectors both have responsibility for surveillance costs. Livestock losses depend on the size of at-risk livestock populations and preventative measures taken, and impact both national economies and households.

The goal of this study was to make the best possible estimate of the burden of rabies, both globally and on a country-by-country basis, by combining all available data sources in a modelling framework that allows us to estimate missing components. We built upon earlier model frameworks [8, 18] to assess the current status of canine rabies globally and provide country-specific estimates of disease burden and associated economic costs. Our model relied on data collected from many different sources including published studies, international databases, market data for vaccine use and expert opinion surveyed for this study. We established relationships between rabies mortality and rabies control measures, which we incorporated into our estimation methods. These relationships indicated how interventions could affect the future burden of disease.

## Methods

### Model Framework

**Fig 1:**
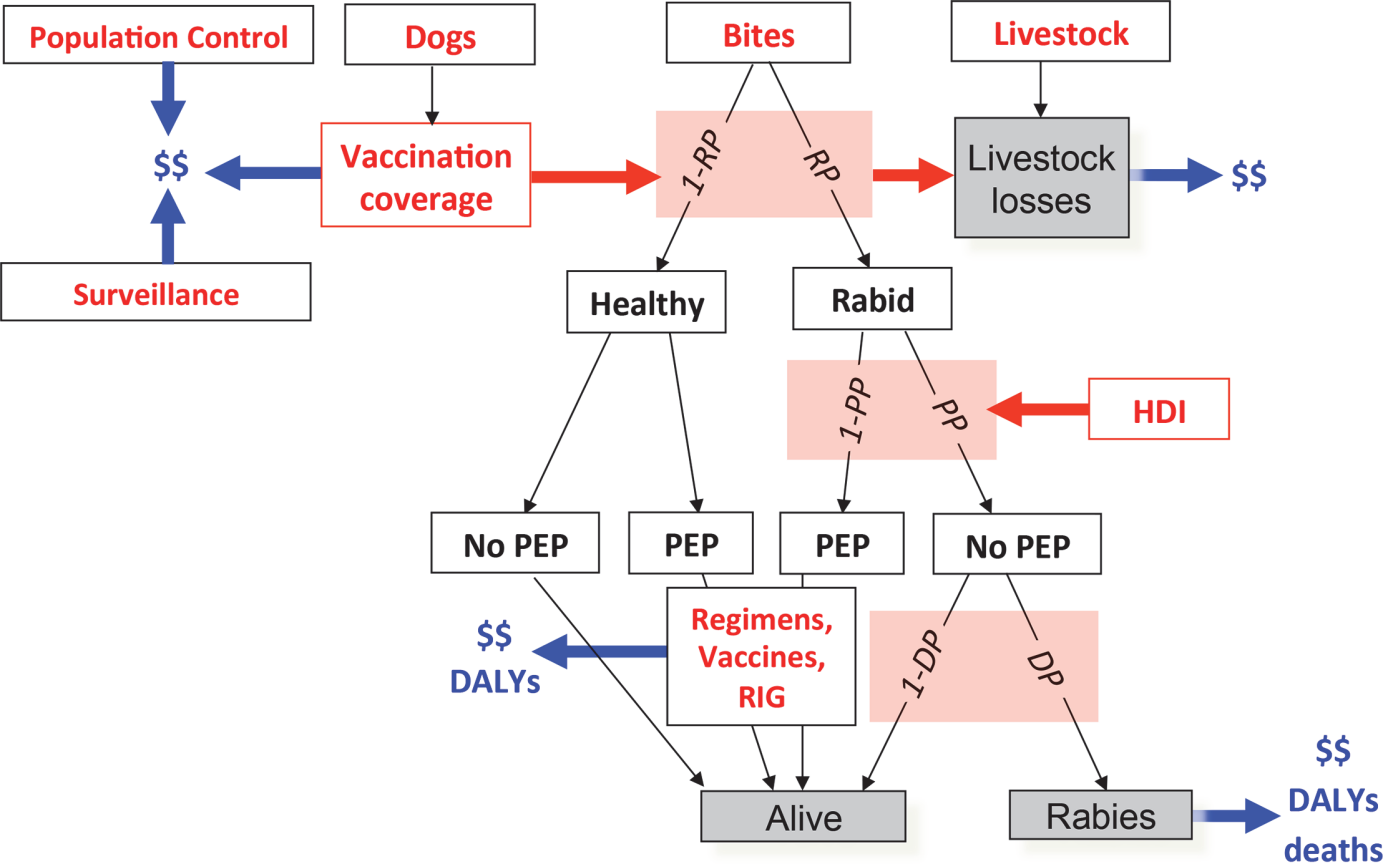
Model framework used in this study for estimating the global burden of canine rabies. Probability steps correspond to the probability that a bite is by a rabid animal (*RP);* that the victim received post-exposure prophylaxis, PEP, (*PP)*; and, in the absence of PEP, that the bite victim developed rabies (*DP)*. Data inputs (Table 1) are shown in red and model outputs in blue. Red arrows show estimated relationships (Fig 2). DALY = Disability-Adjusted Life Year; HDI = Human Development Index. Further details are given in Table 1.

We adapted the probability decision-tree framework developed by Cleaveland *et al*. [8] for Tanzania and used by Knobel *et al*. [18] to estimate the burden of rabies in Africa and Asia. The model uses the product of bite incidence, the probabilities of (i) a biting animal being rabid, *RP*, (ii) a bite victim receiving PEP, *PP*, and (iii) in the absence of PEP, developing rabies, *DP*, to extrapolate human rabies deaths and DALYs. An economic component is included to calculate the costs of rabies prevention and control, such as PEP administration, surveillance and livestock losses from rabies (Fig 1 and Table 1). We parameterized the model using country-specific data or aggregated cluster estimates as described below.

**Table 1:** Data sources and inference methods within framework used for estimating the burden of canine rabies.

Input	Dependencies	Inference method	Data source
Persons seeking PEP for dog bite exposures, *Bites*	None	Cluster values applied where missing data	Literature, surveys with Delphi process
Probability that bite is due to a rabid animal, *RP*	*RP*_*max*_ (in absence of dog vaccination) and dog vaccination coverage, *VC*	ML fit between dog vaccination coverage and dog rabies incidence time series (Fig 2). Extrapolation based on country and cluster estimates of *VC*	*RP*_*max*_ from [23]. Coverage and incidence data from REDIPRA reports and [50].
Vaccination coverage in the dog population, *VC*	None	Cluster values applied where missing data	Literature, surveys and market data, with Delphi process.
Dog population, *Dogs*	Human population data	Reported dog population estimates or projected from dog: human ratios using human population data	UN human population data (esa.un.org/wpp), literature and surveys
Dog *Population Control* (culling and sterilization)	None	Cluster values applied where missing data	Literature and surveys
Laboratory *Surveillance* for human and animal rabies cases	None	Cluster values applied where missing data	Databases (www.who-rabies-bulletin.org/, www.oie.int/wahis_2/public/wahid.php,siepi.panaftosa.org.br), literature and surveys
*Livestock losses* (deaths of cattle, sheep and goats)	*Livestock*, *RP* and *VC*	ML fit between cross-sectional vaccination coverage and livestock incidence data (Fig 2)	Literature [14, 23–25]
Populations of cattle, sheep and goats, *Livestock*	None	FAO database country values	FAO (kids.fao.org/glipha/)
Probability of receiving PEP following exposure by a rabid animal, *PP*	HDI, officially reported deaths and bites	MLE fitted relationship between HDI and probability of receiving PEP (Fig 2)	UN (hdr.undp.org/en/statistics/hdi), literature and surveys
Probability of developing rabies in the absence of PEP following a rabies exposure, *DP*	None	Literature	[15]
*PEP* regimen including whether RIG administered, type of vaccine, route of administration and clinic visits required	None	Literature, surveys and market data	Literature, surveys and market data
Life tables for DALY calculations	None	Global Burden of Disease Study, 2010	[28]
Disability Weightings for DALY calculations	None	Literature	[18]

PEP = post-exposure prophylaxis, HDI = Human Development Index, FAO = Food and Agriculture Organization of the United Nations, REDIPRA = Directors of National Programs to Control Rabies in the Americas, ML = Maximum Likelihood, *Bites* = Bite Incidence (the same annotation is used in Fig 1).

**Fig 2:**
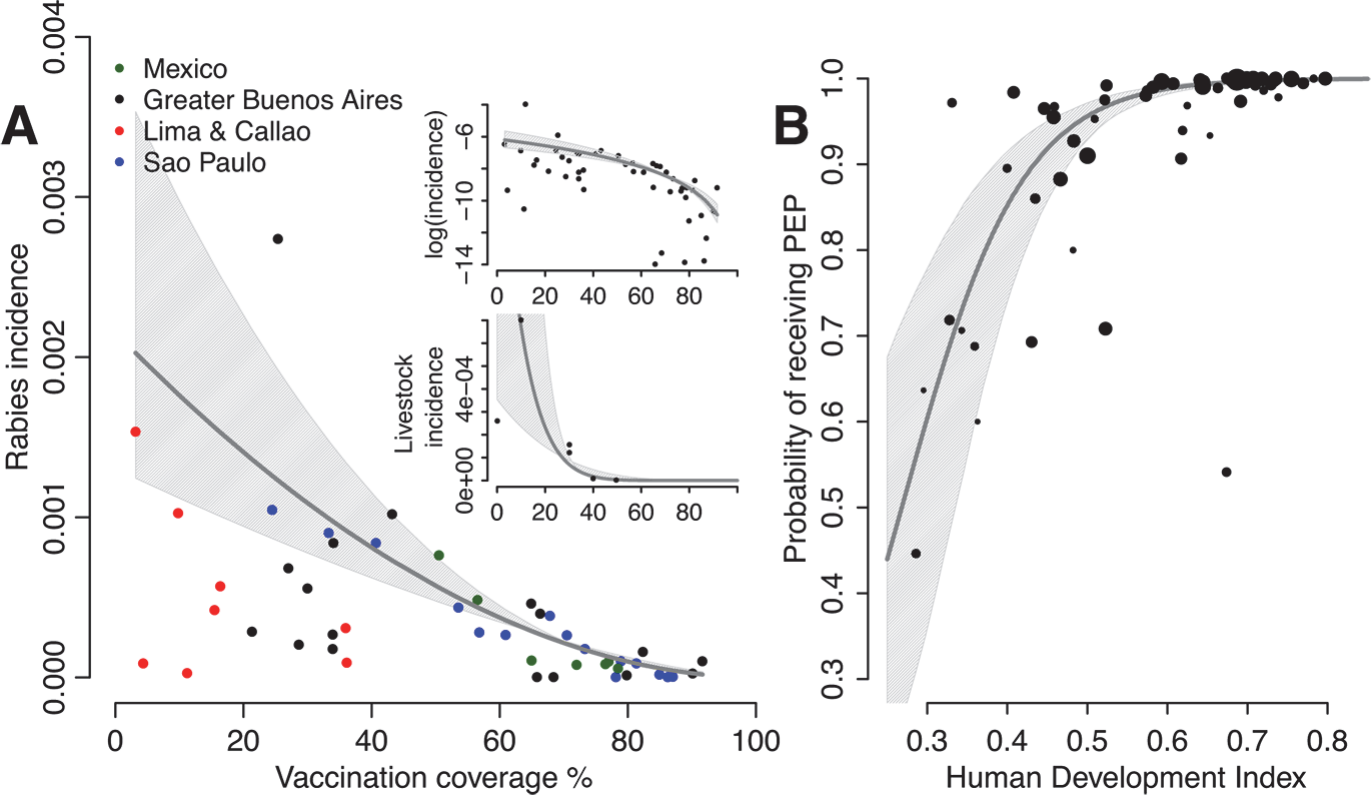
Functional relationships estimated between A) rabies incidence in domestic dogs and average biannual dog vaccination coverage and B) probability of receiving post-exposure prophylaxis (PEP) and the Human Development Index. Upper inset in A shows the relationship on a log scale and the lower inset shows the relationship between rabies incidence in livestock and vaccination coverage in domestic dogs. Grey shading shows the 95% confidence intervals of the fitted relationships.

In endemic areas we assumed that dog rabies incidence depends on vaccination coverage in the dog population, and that the probability that a bite is by a rabid animal depends on incidence. Using concurrent time series from the Americas we identified the best fitting relationship between incidence, *I*, and vaccination coverage, *VC*, estimating the asymptote, *I*_*max*_ and exponent, *S*, of this relationship by maximum likelihood (Fig 2A):

$$I=I_{max}*{\left(1\text{-}VC\right)}^{\text{s}}$$

We assume that the probability that a bite is by a rabid animal is proportional to incidence:

$$RP=RP_{max}*{\left(1\text{-}VC\right)}^{\text{s}}$$

where *RP*_*max*_ (0.74) is the estimated proportion of bites due to rabid animals in countries with negligible vaccination coverage [23]. Using this relationship we generated country and cluster estimates of *RP*. We similarly inferred the relationship between livestock rabies incidence and vaccination coverage, based on cross-sectional data from published studies [14, 23–25] (Fig 2A inset).

We assumed that in the absence of PEP, 19% of victims bitten by rabid dogs develop rabies and die (*DP* = 0.19, see [26]). The probability that a bite victim receives PEP (anti-rabies vaccine, sometimes supplemented by RIG for severe exposures) determines the likelihood of progression to rabies and death, however, we know of only one study quantifying this probability, *PP* [14]. We therefore used an alternative inference method. Reporting of PEP use and of rabies deaths varies according to health infrastructure, however both can be very poor in developing countries. If we assume equivalent reporting rates of rabies deaths, *D*, and PEP use, *T*, we can use the ratio *D*/*T*, as well as the probability that a reported bite was by a rabid dog, *RP*, and the probability of developing rabies in the absence of PEP, *DP*, to calculate the probability that a bite victim receives PEP:

PP=RP*DP/((RP*DP)+D/T)

We examined whether a relationship exists between country economic/welfare measures (the Human Development Index, HDI) and these estimates of the probability of receiving PEP, *PP* (Fig 2B) using a generalized linear mixed-effects model [27] with country as a random effect. We generated country-specific parameter estimates of *PP* and used bootstrap resampling from the fitted mixed model for sensitivity analyses and to generate prediction intervals. We used these parameter estimates together with data on bite incidence (see below) to generate estimates of rabies exposures and deaths.

Disease burden was expressed in terms of standard DALYs and calculated in accordance with methods developed by the World Health Organization (WHO). DALY calculations involve two components: Years of Life Lost (YLL), which, for rabies, captures deaths due to infection, and the Years of Life lived with Disability (YLD), which, for rabies, captures disability following AEs due to use of nerve tissue vaccines, which are still used in some parts of the world. Total YLL due to rabies were estimated using the reference-standard life table from the 2010 Global Burden of Disease Study [28] and the age distribution of rabies cases and exposures from previous research [18](Table 1). We provide burden estimates based on age- and country-specific mortality rates for comparison (S1 Table). The disability weighting used to calculate AEs from nerve tissue vaccines was based on previous research [18]. We explored an additional component in YLD as the anxiety associated with dog bites that may develop into rabies, assuming that the disability level associated with anxiety was 0.108 and that anxiety lasted for 60 days. However, we did not include this in the total disease burden calculation, due to a lack of data to validate these assumptions. The data, parameter values and code to replicate all the analyses are provided in the Supporting Information (S1 compressed file archive).

### Clustering of Countries

We aggregated countries into clusters on the basis of similar rabies epidemiological situations, socioeconomic conditions and geographical proximity. Countries were classified as canine rabies-free based on historical freedom or literature reporting canine rabies elimination. Oceania, Western Europe, the US and Canada and canine rabies-free countries in Asia (Japan, Malaysia, Singapore, and Republic of Korea) comprised four canine rabies-free clusters that were not included in further analysis. The disease burden was estimated in endemic countries within clusters. Where possible, country level data was used to parameterize the model, but, in the absence of country-specific information, we applied the average estimate from countries within the cluster.

### Data Sources

Data were obtained from:

1. Surveys involving the medical, veterinary and laboratory sectors. The information gathered included reported clinical and laboratory-confirmed rabies cases in humans and animals, protocols for and expenditure related to PEP and costs of control efforts (vaccinating, sterilizing and killing dogs). Surveys were translated into French, Spanish, Portuguese and Russian and made available online. We solicited responses from country representatives, particularly those attending regional rabies meetings or identified through regional networks (such as the Southern and Eastern Africa Rabies Group, SEARG, and Directors of National Programs to Control Rabies in the Americas, REDIPRA [19]), responsible for reporting on rabies surveillance and diagnosis in their countries. Data was collected from 136 respondents (spanning all sectors and 45 countries). These surveys provided valuable data on rabies prevention and control practices, including PEP protocols and unit costs. However, most quantitative data on incidence were incomplete and we therefore used published data instead (see point 2).
2. An extensive literature search for estimates of human and animal rabies, dog bite incidence, control efforts and associated economic costs. We searched Web of Knowledge and PubMed for publications from 2000 to 2013 using ‘rabies’ AND ‘dogs’ as key words and resulting papers were reviewed to determine their relevance. In addition to scientific publications within the standard search, we collated technical reports and presentations from regional meetings, soliciting data through members of the Global Alliance for Rabies Control Partners for Rabies Prevention (PRP, rabiesalliance.org/about-us/partners). We identified 551 articles addressing canine rabies and its control, but useable quantitative information was only available from a smaller subset (113, see the supporting bibliography, S1 text), with considerable underreporting evident in official reports of bite incidence and rabies deaths from low and middle-income countries. We used estimates of bite incidence from empirical studies involving active surveillance and only incorporated official data where no other sources were available and where these were deemed valid by the PRP group. We used the most recent data available since 2000. Searches were last updated on 1 June 2013.
3. International databases for country-specific estimates of human populations, demographic rates, economic indicators and livestock (detailed in Table 1). GDP and 2010 exchange rates were from the International Monetary Fund (IMF) databases; 2010 human population estimates and HDI estimates were from the United Nations; health costs were from the WHO (WHO-CHOICE database; CHOosing Interventions that are Cost-Effective), and livestock populations were from the Food and Agriculture Organization (FAO) Global Livestock Production and Health Atlas. The World Organization for Animal Health (OIE) World Animal Health Information System (WAHID) and surveillance databases for specific regions were used to obtain recent country-specific reports of laboratory confirmed rabies cases and diagnostic tests performed.
4. Estimates of regional markets for dog rabies vaccines from Merial and for human post-exposure vaccines and RIG from Sanofi Pasteur. These were used to validate dog vaccination coverage estimates or to provide estimates of coverage for areas where no other source of information was available. If market estimates differed from other sources by >10%, experts were consulted (amongst the PRP group) and a 2-round delphi process [29] used to obtain consensus on values used in the analysis.

### Economic Costs

The economic cost of deaths due to rabies was estimated using the human capital approach based on productivity losses. For each rabies death, the number of discounted life years lost was based on the age distribution of rabies deaths and remaining life expectancy using the reference-standard life table from the 2010 Global Burden of Disease Study [28]. Productivity losses were calculated by weighting the life years lost by the country-specific GDP per capita without discounting. Estimates were also calculated using age-weighting and time discounting for comparison with other studies. Time lost by victims and accompanying care-givers (assuming all minors were accompanied by one adult) whilst seeking PEP was incorporated into economic losses. Country estimates of unit costs for delivering PEP, dog vaccination and surveillance were largely obtained from surveys (detailed in 1). The total economic cost of rabies was obtained by combining data on unit cost per case, livestock losses, and costs of control and prevention.

We updated reported costs to 2010 US dollars (USD) using IMF statistics. We corrected for international differences in medical costs using the WHO-CHOICE database. Indirect costs were corrected for differences in income using the ratio of income per capita (expressed in International dollars, I$) calculated using IMF statistics. Direct non-medical costs were corrected only for differences in purchasing power.

Livestock losses due to dog rabies were extrapolated from the inferred relationship between coverage and incidence (Fig 2A inset) and using livestock population estimates from FAO. Incidence of rabies in cattle was multiplied by the cost per head of cattle. Using the fitted relationship between dog vaccination coverage and reported rabies incidence in livestock [14, 23, 25, 30] described above, and country and cluster values for dog vaccination coverage we estimated livestock losses. We converted the costs of different livestock into the costs of cattle using FAO livestock unit measures [31].

### Sensitivity Analyses

Uncertainty was modelled by drawing from distributions for each parameter estimate.

Bite incidence and dog vaccination coverage were modelled using triangular distributions, with maxima and minima set according to cluster ranges agreed from the 2-round Delphi process. Uncertainty in PEP probability was modelled using bootstrap resampling from the fitted mixed model described above. Uncertainty in the other probabilities (*RP*, and *DP*) was modelled using permutation-based resampling based on the original binomial sample. The range of variation of the results of our analysis was assessed using 1,000 Monte Carlo simulations. Parameters that varied across countries or clusters used the same quantile draw globally for each realization.

## Results

### Data Validation and Model Fitting

Rabies incidence in dogs was best described by the fitted model (Fig 2A):

$$I=0.002*{\left(1\text{-}VC\right)}^{1.9}$$

with dog vaccination coverage corresponding to the average from the previous two years. We used this relationship to infer the probability that a bite was due to a rabid animal, *RP*. We inferred a similar relationship between rabies incidence in livestock, *IL* and dog vaccination coverage (Fig 2A inset):

$$IL=0.0017*{\left(1\text{-}VC\right)}^{9}$$

We found a strong significant relationship between inferred probability of receiving PEP and both country GDP and HDI (p<0.001). We used the relationship with HDI, which better captures inequalities within countries (Fig 2B) to generate estimates of the probability of receiving PEP, *PP*. The parameter estimates *RP* and *PP* are detailed by cluster in Table 2 and by country in S1 Table. Using these relationships and the data described above, we implemented the probability decision tree model to generate burden estimates.

**Table 2:** Estimates of rabies deaths, exposures, PEP use, prevented deaths, DALYs (due to rabies and to NTVs), and average dog vaccination coverage, probability that a dog is rabid *(RP)*, and probability of receiving PEP (*PP*) by cluster in canine rabies endemic regions.

Cluster	Deaths [95% CI]*	Exposures	PEP	Prevented deaths	YLL rabies	DALY NTVs	DALYs total	Dog Vaccination Coverage %	*RP*	*PP*
Asia 2	5,423 [1–10]	766,842 [238–1313]	1,143,377 [418–2076]	140,277 [44–275]	338,639 [76–635]	18,376 [4–19]	357,015 [80–655]	9%	0.671	0.973
Asia 3	2,438 [1–13]	244,767 [144–1139]	335,740 [220–1612]	44,068 [26–227]	152,263 [69–799]	8,538 [6–54]	160,801 [75–853]	5%	0.673	0.952
Asia 4	265 [0–1]	262,841 [179–917]	862,641 [502–2123]	49,675 [32–191]	16,521 [10–83]	[0–0]	16,521 [10–83]	36%	0.337	0.995
China	6,002 [1–11]	8,318,530 [1514–10993]	14,943,066 [3811–19223]	1,574,518 [313–2345]	374,851 [60–674]	[0–0]	374,851 [60–674]	14%	0.555	0.996
India	20,847 [7–55]	4,581,603 [1553–9619]	8,209,470 [2832–16149]	849,658 [293–1974]	1,301,865 [377–3436]	[0–0]	1,301,865 [377–3436]	15%	0.545	0.976
Indonesia	197 [0–3]	108,322 [118–1428]	242,725 [271–3013]	20,384 [22–293]	12,311 [12–198]	[0–0]	12,311 [12–198]	24%	0.442	0.972
North Africa	1,971 [1–7]	195,237 [61–404]	403,632 [120–699]	35,124 [11–80]	123,074 [38–467]	[0–0]	123,074 [38–467]	10%	0.562	0.892
Congo Basin	7,196 [4–16]	119,707 [105–186]	116,433 [86–189]	15,548 [10–29]	449,382 [244–1031]	[0–0]	449,382 [244–1031]	9%	0.701	0.829
West Africa	6,005 [3–15]	258,341 [214–431]	350,374 [317–566]	43,080 [30–82]	375,023 [206–971]	[0–0]	375,023 [206–971]	10%	0.718	0.837
SADC	6,330 [2–27]	274,041 [118–818]	517,409 [200–1227]	45,738 [19–147]	395,297 [155–1702]	2,867 [2–11]	398,164 [157–1713]	23%	0.661	0.913
Andean	18 [0–0]	40,950 [12–93]	168,104 [99–416]	7,763 [2–20]	1,108 [0–3]	473 [0–1]	1,582 [0–4]	57%	0.194	0.997
Brazil	16 [0–0]	34,255 [18–46]	427,604 [282–485]	6,492 [3–10]	1,023 [0–2]	[0–0]	1,023 [0–2]	69%	0.080	0.997
Caribbean	137 [0–0]	21,133 [16–28]	73,557 [65–82]	3,878 [2–6]	8,581 [4–17]	[0–0]	8,581 [4–17]	41%	0.191	0.992
Central America	8 [0–0]	8,272 [4–44]	128,538 [88–706]	1,564 [1–9]	472 [0–3]	23 [0–0]	495 [0–3]	58%	0.166	0.994
Southern Cone	3 [0–0]	18,090 [3–40]	37,854 [31–80]	3,434 [1–8]	189 [0–1]	81 [0–0]	270 [0–1]	52%	0.176	0.998
Eastern Europe	31 [0–0]	65,538 [19–138]	308,522 [96–596]	12,421 [4–29]	1,948 [0–5]	[0–0]	1,948 [0–5]	62%	0.218	0.999
Eurasia	1,875 [1–6]	259,650 [129–625]	672,177 [353–1531]	47,458 [23–127]	117,116 [46–368]	[0–0]	117,116 [46–368]	22%	0.474	0.980
Middle East	229 [0–1]	116,785 [55–192]	233,883 [116–359]	21,960 [10–41]	14,310 [6–39]	[0–0]	14,310 [6–39]	32%	0.476	0.991
**TOTAL**	58,991 [21–167]	15,694,905 [4501–28455]	29,175,105 [9907–51132]	2,923,041 [846–5892]	3,683,974 [1304–10433]	30,359 [12–86]	3,714,333 [1316–10519]			

Estimates by country are provided in S1 Table including which cluster countries were assigned to.

*95% Confidence Intervals (Thousands)

PEP = post-exposure prophylaxis, YLL = Years of life lost, DALY = Disability Adjusted Life Year, NTVs = adverse events from Nerve Tissue Vaccines. Asia 4 comprises the Philippines, Sri Lanka, Thailand (High PEP use); Asia 3 comprises Bhutan, Nepal, Bangladesh, Pakistan (Himalayan region); Asia 2 comprises Cambodia, Myanmar, Laos, Vietnam and Democratic People’s Republic of Korea; SADC comprises countries in the Southern African Development Community, Eurasia comprises Afghanistan, Kazakhstan, Kyrgyzstan, Mongolia, the Russian Federation, Turkmenistan, Tajikistan, and Uzbekistan.

### Human Rabies Deaths and DALY Estimates

**Fig 3:**
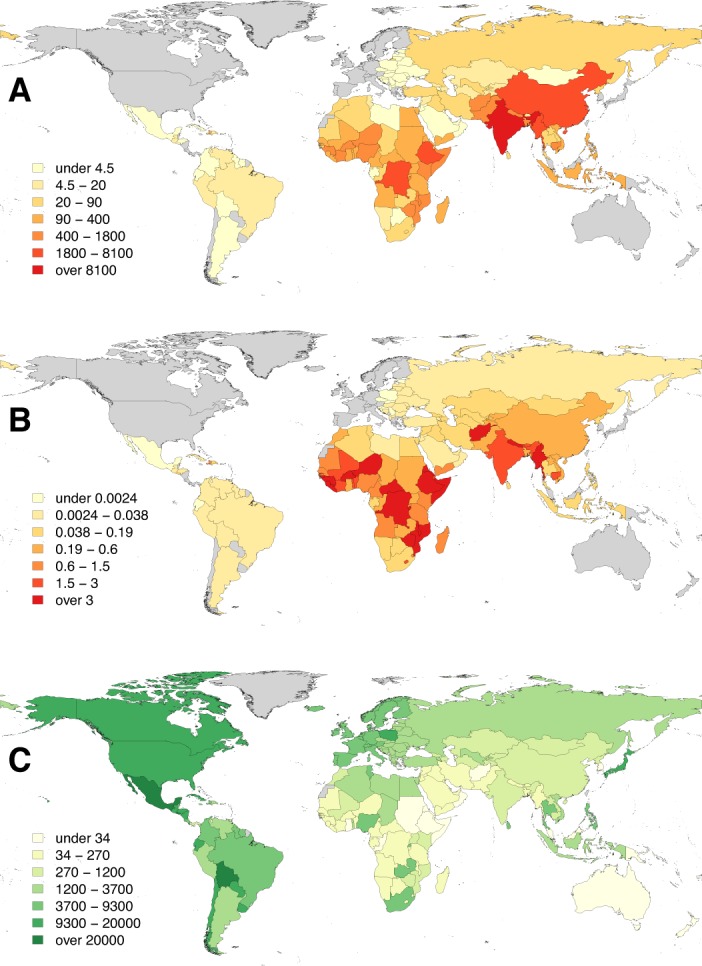
The distribution of the global burden of rabies: A) human rabies deaths, B) per capita death rates (per 100,000 persons), and C) expenditure on dog vaccination (per 100,000 persons). Countries shaded in grey are free from canine rabies.

**Fig 4:**
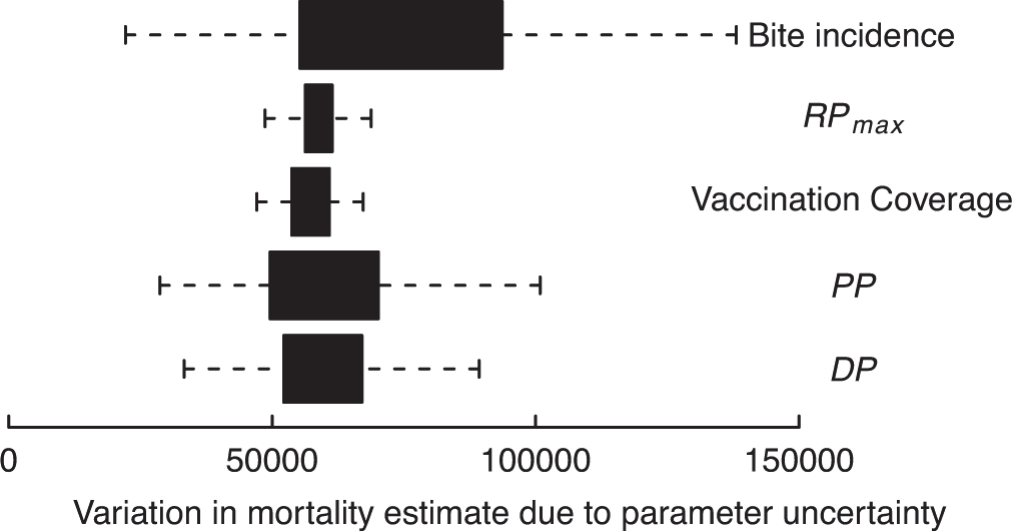
Model sensitivity to parameter uncertainty. PEP = post-exposure prophylaxis.

Results of predicted rabies mortality, morbidity and DALYs are provided by cluster in Table 2 and by country in S1 Table. We estimated that around 59,000 [95% CIs: 25,000–159,200] human rabies deaths occur annually globally, with the vast majority of these in Africa (36.4%) and Asia (59.6%). Less than 0.05% of estimated deaths occurred in the Americas [182, 95% CIs: 84–428], of which over 70% were from Haiti. India, with 35% of human rabies deaths, accounted for more deaths than any other country, but the estimated per-person death rate was highest in the poorest countries in sub-Saharan Africa. The global distribution of estimated human deaths and death rates due to rabies is illustrated in Fig 3. The parameters that had the greatest impact on variation in estimated human rabies mortality were bite incidence, followed by the probability of receiving PEP and the probability of developing rabies after a rabid animal bite in the absence of PEP (Fig 4).

Globally, around 3.7 [1.6–10.4] million DALYs were estimated to be lost due to rabies, with over 95% lost in Africa (36.2%) and Asia (59.9%) and less than 0.5% (11,950 DALYs) in the Americas. The vast majority (>99%) of DALYs lost (3.68 million) were due to the premature death of rabies victims (YLL). A very small part (0.8%) of the DALY score (30,400) was due to AEs (in terms of YLD) of outdated, mostly locally produced, nerve tissue vaccines still in use in at least 10 countries in 2010. Anxiety due to suspect rabid dog bites potentially accounts for a substantial burden (518,000 DALYs in terms of YLD), although we do not include this component in our total estimate due to a lack of data to validate assumptions about disability weighting (0.108) and its application (60 days for true exposures).

### Economic Costs

**Fig 5:**
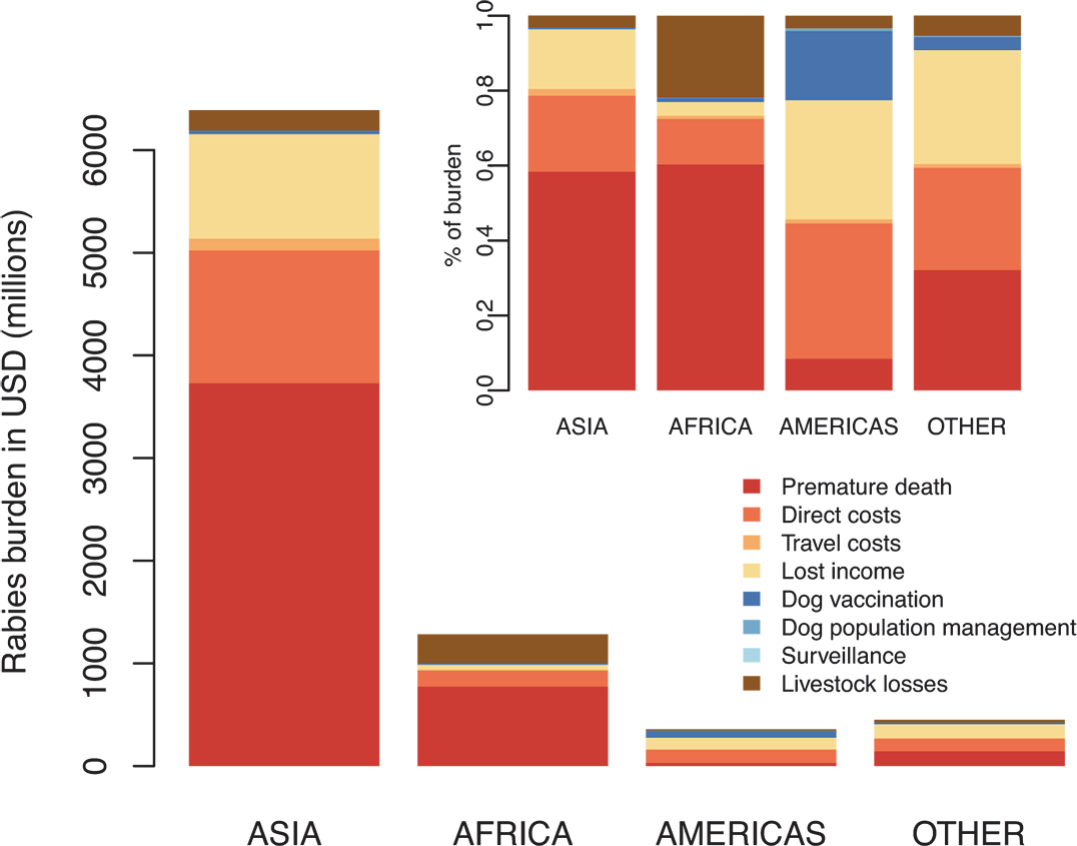
Division of costs associated with rabies, prevention and control across sectors by region. Inset shows proportional expenditure in different regions. The breakdown of costs by cluster is given in S1 Fig and Table 3 and detailed by country in S1 Table.

The overall economic costs of canine rabies were estimated as 8.6 billion USD (95% CIs: 2.9–21.5 billion). These costs were mainly due to productivity losses from premature deaths (2.27 billion USD), direct expenditure on PEP (totalling 1.70 billion USD) and lost income whilst seeking PEP (1.31 billion USD). However, there was considerable variation in the breakdown of costs by region (Fig 5): the largest proportion of costs was due to premature death in Asia and Africa; much less was lost due to PEP (direct costs, and from travel and lost income whilst seeking PEP) in Africa compared to Asia and the Americas, and in the Americas a large proportion of costs were due to dog vaccination. Livestock deaths amounted to 512 million USD per year, with major losses in African countries with livestock-dependent economies (e.g. Ethiopia, Sudan, and Tanzania) and in more populous countries in Asia (China, India, Bangladesh and Pakistan). Table 3 provides a breakdown of estimated costs and a country breakdown is given in S1 Table.

**Table 3:** Breakdown of economic costs of rabies by cluster in thousands of USD.

Cluster	Direct Costs	Travel costs	Lost Income	Productivity losses from premature death	Livestock losses	Dog vaccination	Dog population management	Surveillance
Asia 2	75.15	15.96	20.90	252.298	2.073	0.074	24.163	0.000
Asia 3	16.67	4.42	7.08	104.774	0.564	0.214	65.706	0.042
Asia 4	41.81	2.32	33.26	45.658	11.248	0.123	1.728	0.021
China	648.27	49.25	807.32	1,642.646	4.235	0.195	40.777	0.046
India	491.23	42.60	138.03	1,646.650	9.050	0.417	62.348	0.002
Indonesia	21.18	0.95	10.03	37.123	6.384	0.811	1.717	0.000
North Africa	38.11	1.45	19.70	106.002	2.756	1.013	89.661	0.040
Congo Basin	14.47	1.28	2.33	154.424	0.481	0.003	19.670	0.001
West Africa	48.53	2.94	5.37	313.348	6.684	0.026	60.086	0.004
SADC	55.00	4.81	19.61	199.579	4.600	0.263	110.129	0.079
Andean	32.00	1.24	27.26	7.867	10.753	0.396	3.130	0.014
Brazil	45.37	1.82	63.36	11.070	16.620	0.342	0.007	0.288
Caribbean	11.66	0.24	2.43	7.702	2.575	0.113	0.296	0.006
Central America	34.13	0.49	11.74	1.873	31.308	0.809	0.001	0.020
Southern Cone	6.18	0.13	9.00	1.730	4.710	0.521	8.753	0.007
Eastern Europe	51.09	1.84	41.88	18.350	10.460	0.053	0.627	0.062
Eurasia	28.93	2.17	68.66	89.932	4.451	1.413	13.758	0.083
Middle East	42.58	0.35	26.06	35.907	0.592	0.128	9.543	0.025
**TOTAL**	1702.35	134.28	1314.01	4676.93	129.55	6.91	512.10	0.74

Estimates by country are in S1 Table including which cluster countries were assigned to. Asia 4 comprises the Philippines, Sri Lanka, Thailand (High PEP use); Asia 3 comprises Bhutan, Nepal, Bangladesh, Pakistan (Himalayan region); Asia 2 comprises Cambodia, Myanmar, Laos, Vietnam and Democratic People’s Republic of Korea; SADC comprises countries in the Southern African Development Community, Eurasia comprises Afghanistan, Kazakhstan, Kyrgyzstan, Mongolia, the Russian Federation, Turkmenistan, Tajikistan, and Uzbekistan.

Globally, over 70% of the estimated economic burden was societal (from premature deaths and losses from seeking PEP); 20% fell to the medical sector or to bite victims (direct costs) and ~8% to the veterinary sector or directly to communities, from livestock losses and control interventions (dog vaccination and population management i.e. culling and/or sterilization/ birth control). Only around 0.01% of costs were from laboratory-based surveillance. The breakdown of costs by region varied dramatically (Figs 5 and S1 and S1 Table gives country breakdown). Dog vaccination accounted for less than 1.5% (~$130 million) of the economic burden. In the Americas, average per capita expenditure on dog vaccination was approximately $0.11, amounting to almost 20% of the economic burden. In most other endemic low-income countries, per capita expenditure on dog vaccination was negligible (less than $0.02 or <2% of the economic burden respectively, Figs 3C and 5 inset).

Unit costs differed greatly between countries both for dog vaccination (for example costing $6-7/dose in some West African countries, $1/dose in Laos, $0.45/dose in the Philippines, $0.5/dose in Chad and $0.2–0.3 in Tanzania) and human PEP (range: $11–150 per dose), as well as the regimens and types of vaccines and RIG used (compressed file archive). Most countries with a high disease burden reported negligible use of RIG. Countries with more substantial RIG use were in Eastern Europe, North Africa and a few Asian countries (Sri Lanka, Thailand and the Philippines). A few countries reported use of NTVs, notably Ethiopia, several countries in Latin America (Peru, Venezuela, Bolivia, Honduras and Argentina), Myanmar and Pakistan, with Bangladesh discontinuing use of NTVs in 2011 [1] (though were classified as using NTVs for the year 2010). Only a few countries reported widespread ID vaccination, including the Philippines, Sri Lanka, Thailand, and to a much lesser extent in India.

## Discussion

This study highlights that the mortality risks and per capita rabies burden fall disproportionately upon the poorest regions of the world, with impacts on local communities, public health sector budgets and livestock economies. Mortality and loss of economic productivity due to premature death are the most serious effects of canine rabies. Highest mortality rates occur in areas with limited dog vaccination, where PEP is the only lifeline for at-risk populations, yet PEP supply and distribution systems are wholly inadequate in many of these places and often very costly. PEP costs, the second largest component of the economic burden, could be reduced in many areas through more judicious and cost-effective administration. The methods developed here shed light on important gaps in knowledge, provide a preliminary picture of the distribution of the rabies burden by country and underline the lack of investment in rabies control and prevention measures. Improved surveillance and reporting of bites and rabies cases is needed, both for better burden estimates, and most importantly to monitor the impacts of control efforts.

We estimate that annually canine rabies causes around 59,000 deaths and 3.7 million DALYs, which is considerably higher than previous estimates [18], however this is largely due to methods. In our study we did not apply time discounting or age-weighting to calculate DALYs for consistency with the Global Burden of Disease (GBD) study 2010 [32, 33]. Our DALY estimates are correspondingly higher than the GBD study as a result of our higher estimates of mortality. Applying 3% discounting and age-weighting we estimated slightly higher but more consistent DALY estimates to the Knobel *et al*. 2005 study [18]. S1 Table shows differences in DALY estimates due to the life table used and age-weighting and time discounting. We estimated a much higher economic burden of around 8.6 billion USD annually, compared to 583.5 million USD reported by Knobel *et al*. [18]. These differences are due to the inclusion of lost income from premature deaths and from all countries (not just those of Africa and Asia), in addition to a major increase in estimated direct PEP costs from 300 million USD [18] to 1.7 billion USD. Reasons contributing to this increase include higher prices and increased availability of cell-culture vaccines compared to NTVs, now discontinued in most endemic areas. Furthermore, we estimated higher bite incidence and PEP use (29.2 versus 4.3 million PEP delivered, respectively), based on published data, with over 10 million PEP delivered in China alone [34].

According to our estimates, India, the world’s second most populous country (with close to 18% of the global population) accounts for over 35% of the global rabies burden (approximately 20,800 deaths). This is broadly consistent with a recent verbal autopsy study, that estimated ~12,700 deaths from furious rabies alone in India [12]. Generally, our country estimates are in line with active surveillance studies [10, 13]. However, our estimates of deaths and DALYs are considerably higher than the GBD study, which attributed only 26,400 deaths and 1.46 million DALYs worldwide to rabies in 2010 [32, 33]. The GBD study drew upon officially recorded data for rabies, which grossly underestimate the disease burden due to extensive underreporting from countries where rabies is most prevalent. A large proportion of rabies deaths are misdiagnosed in areas with high general mortality [16]. Hospitalization provides little palliative care and death is inevitable, therefore many victims, particularly those from poor communities, either do not attend a facility or do not stay until death. Most victims (>75%) die at home [10,11,13], and these deaths are absent from official records. Furthermore, few clinical rabies diagnoses will be made from verbal autopsies unless interviewers probe for a history of a dog bite (as in [12]). Therefore even in countries where rabies is notifiable, many rabies deaths are not recorded, and burden estimates must therefore rely on predictive approaches. Our model provides a point of comparison for individual countries, but, due to the nature of extrapolating across large and heterogeneous populations, inaccuracies are likely and active surveillance studies are warranted.

A major question is how to quantify anxiety associated with a life-threatening bite from a rabid animal. Previous burden estimates have ignored this component. We were also unable to find empirical evidence to validate a disability weighting. However, using assumptions agreed upon by the PRP group, we show that anxiety could be substantial (>10% of the total burden, ~518,000 DALYs), but research is needed to validate this weighting and its application to bite victims. In our study, AEs from NTV use account for a very small proportion of DALYs (0.8%, 30,400 DALYs), and this has declined (44,900 DALYs in 2005 [18]) due to the discontinuation of NTVs in most countries.

Lost productivity due to premature death ($4.7 billion 55.2%) was the largest component of the economic burden followed by direct costs of PEP ($1.7 billion, 20.1%). Investment in PEP has reduced rabies deaths in some countries, but at a high cost [35], whilst there has been very little investment in dog vaccination (Figs 3 and 5). More judicious administration of PEP could substantially reduce PEP costs (as indicated by the divergence of estimates of PEP use and prevented deaths detailed in Table 2). For example, >1 million PEP are delivered annually in the Americas [36], but most are precautionary for healthy animal bites. Most countries use IM delivery of PEP, but substantial savings (>60%) could be achieved globally by switching to the more cost-effective ID route as recommended by WHO [1]. Indirect costs of seeking PEP (1.84 billion, both travel and lost income) are a major cost to households of exposed individuals. This is a particular problem in rural areas, since PEP is typically only available in urban centres (sometimes only capital cities [10]). Recent improvements in PEP provision have reduced mortality in some countries [13, 37] and should be considered more widely.

Vaccination of dogs, the proven way of preventing human exposures and eliminating the disease at source, comprised a very small proportion of the economic burden (<1.5%). Outside North America and Europe, a large investment in dog vaccination has only been sustained in one region (~$0.11/person/year in the Americas). The result is that the rabies burden in the Americas is very small (<200 deaths per year across the continent, mostly in Haiti), in contrast to other countries where rabies is endemic and expenditure on dog vaccination is negligible (<$0.02/person/year). Unlike the international government-coordinated control effort across Latin America, many developing countries have relegated rabies control and prevention to the private sector with no regulatory requirements or incentives (for example, as part of structural adjustment programmes in sub-Saharan Africa). As a result, rabies has been neglected in comparison to economically important livestock diseases. Generally, medical sector costs were much higher than veterinary costs (Fig 5), but investment in dog vaccination could bring down costs to the medical sector, demonstrating the need for intersectoral coordination [38, 39]. The World Bank has been supporting the strengthening of veterinary services (e.g. through the OIE Performance of Veterinary Services pathway) and considering zoonoses prevention and control as a ‘public good’, but resources are still lacking. Standardization of vaccine procurement is greatly needed to assist poorer countries to implement mass vaccinations, given the wide variation in vaccine prices shown by our study. Vaccine banks such as those administered by OIE could have a pivotal role to play.

Livestock losses are a relatively small component of the global economic burden (6%), but represent an important cost to impoverished and livestock dependent communities, particularly in Africa [40]. Our estimates should be considered on the low side due to the limited data, drawing from only a few cross-sectional studies [14, 23, 25, 30], including laboratory confirmed cases, which underestimate the true burden [24]. Better reporting of livestock cases (suspect and confirmed) and further active surveillance studies are therefore necessary.

There are number of limitations to our study. The most critical relate to uncertainty surrounding parameter estimates, particularly in relation to bite incidence (Fig 4). A Bayesian Hierarchical approach could better incorporate uncertainties, but estimates will be constrained by the data scarcity and quality. By fitting the relationship between vaccination coverage and dog rabies incidence phenomenologically, using longitudinal time series, the effect of differences in surveillance quality between locations was reduced. High turnover of dog populations means that single vaccination campaigns have short-lived impacts, whereas sustained campaigns progressively reduce disease incidence [3, 41]. Using lagged average coverage from consecutive campaigns improved the model fit and meant estimates were less subject to stochastic fluctuations (Fig 2). However, we had little power to estimate incidence at negligible coverage levels due to large multiannual epidemic fluctuations, compounded by limited surveillance. Consistency in estimates of the basic reproductive ratio for canine rabies [3, 42] provides reassurance for extrapolations, but localized heterogeneities and landscape and demographic characteristics will influence vaccination impacts. Improved surveillance should enable future use of more mechanistic dynamical models [43]. Nonetheless, the direct relationship between dog vaccination and disease incidence provides a logical means of comparison that frames the problem in terms of investment in disease control and prevention.

The proportion of reported bites due to suspect rabid animals contributes uncertainty to our results (Fig 4), and varies according to rabies incidence and treatment-seeking behaviour (Fig 2). But we were unable to find data to classify this systematically. By assuming that the maximum probability that a bite is by a rabid animal was 0.74 in countries with negligible vaccination coverage [23], we set an upper limit on the mortality burden, with reduced mortality rates in countries with higher coverage. More generally, major uncertainties in treatment-seeking behaviour, PEP availability and dog bite incidence limited the accuracy of our estimates. For example, little is known about the variation in PEP seeking in different socio-economic and cultural settings. However, cumulative evidence, confirmed by responses to our questionnaire, shows that PEP accessibility is very poor, often restricted to capital cities in the poorest countries [10], whilst in richer countries, or where PEP is provided free-of-charge, PEP is sought more readily [24, 44].

We ignored mortality and costs due to imported cases in rabies-free countries, which can be individually expensive [45], but are negligible compared to endemic rabies. Our use of average per capita GDP will mean that productive losses are overestimated, as rabies disproportionately affects impoverished communities. A further limitation of our study is that the burden is not broken down between urban and rural areas, due to a lack of data. However, dog vaccinations are implemented mostly in urban areas, which are easiest to access; dog:human ratios are typically higher in rural areas [46–48]; and PEP access is best in capital cities. Hence most rabies cases are expected to be from rural areas [18]. Finally, our estimates do not include the impacts of wildlife-transmitted rabies (from terrestrial wildlife and bats maintaining rabies virus transmission independently from domestic dogs). However, as canine rabies accounts for well over 95% of all human cases, our estimates are expected to be close to the overall rabies mortality burden globally. On the other hand, livestock losses due to wildlife rabies (for example, vampire bat rabies in the Americas [1]), will add substantially to the economic burden of rabies in certain parts of the world.

This study demonstrates that the global burden of canine rabies is substantial, even though the disease is entirely preventable. Success in tackling the problem is contingent on investment in dog rabies control, which we show has been severely lacking. Long-term mass dog vaccination efforts could reduce medical sector and societal costs, and elimination is feasible with currently available methods [40, 49], however innovative financing models are required to overcome institutional barriers.

## Supporting Information

S1 Text Supporting bibliography. (DOCX)

S1 Table Estimates by country of rabies deaths, exposures, PEP use, prevented deaths, dog vaccination coverage, probability that a dog is rabid *(RP)*, of bite victims receiving PEP (*PP*), DALYs, costs and 95% confidence intervals of estimates. Clusters to which countries are assigned are shown and inputs used for estimating parameters including the human development index and whetehr a country s rabies-free or endemic (RISK). Estimates of years of life lost (YLL) and DALYs (due to rabies and to adverse events from the use of nerve tissue vaccines) are shown under different assumptions (estimates under the assumption of no time discounting or age-weighting should be directly comparable to the 2010 Global Burden of Disease study).
(XLSX)

S1 Fig Division of costs associated with rabies, prevention and control across sectors by cluster. Inset shows proportional expenditure in different clusters. Full details of countries by cluster are given in S1 Table. Asia 4 comprises: Philippines, Sri Lanka, Thailand (High PEP use); Asia 3 comprises Bhutan, Nepal, Bangladesh, Pakistan (Himalayan region); Asia 2 comprises Cambodia, Myanmar, Laos, Vietnam and Democratic People’s Republic of Korea; SADC comprises countries in the Southern African Development Community, Eurasia comprises Afghanistan, Kazakhstan, Kyrgyzstan, Mongolia, the Russian Federation, Turkmenistan, Tajikistan, and Uzbekistan.
(EPS)

S1 Dataset Model code and input data files including references, rationale and detail of Delphi process. The *code* folder contains seven R scripts: *burden_model*.*R* runs the model using data compiled in *burden_1*.*R*, after estimating parameters using: *FitCovInc*.*R*, *FitPP*.*R*, and creating Fig 2 (*RabiesBurdenFig2*.*R*). The script *burden_results*.*R* summarizes findings using the output of *burden_model*.*R* and *burden_sensitivity*.*R* runs the sensitivity analyses. The *data* folder contains 12 csv files called by the R code for the analyses, and one excel file (Vet.xlsx) with additional details about the data sources in vcountry2.csv and vcluster2.csv and with Delphi process estimates for dog vaccination coverage. Data sources are detailed in the relevant data sources and the details of the sources of data used in the analysis are in the supporting bibliography, S1 text.
(ZIP)
