# Correction: Estimating the Global Burden of Endemic Canine Rabies

**DOI:** 10.1371/journal.pntd.0003786

**Published:** 2015-05-11

**Authors:** Katie Hampson, Laurent Coudeville, Tiziana Lembo, Maganga Sambo, Alexia Kieffer, Michaël Attlan, Jacques Barrat, Jesse D. Blanton, Deborah J. Briggs, Sarah Cleaveland, Peter Costa, Conrad M. Freuling, Elly Hiby, Lea Knopf, Fernando Leanes, François-Xavier Meslin, Artem Metlin, Mary Elizabeth Miranda, Thomas Müller, Louis H. Nel, Sergio Recuenco, Charles E. Rupprecht, Carolin Schumacher, Louise Taylor, Marco Antonio, Natal Vigilato, Jakob Zinsstag, Jonathan Dushoff, Global Alliance for Rabies Control Partners for Rabies Prevention

There are a number of errors in Table 3. The table legend should read: Breakdown of economic costs of rabies by cluster in millions of USD. The headings for columns six, seven, and eight are incorrect. They should be in the following order: Dog vaccination, Dog population management, Livestock losses. Please see the correct Table 3 below.

**Table 3:** Breakdown of economic costs of rabies by cluster in millions of USD. Estimates by country are in Table S1 including which cluster countries were assigned to. Asia 4 comprises the Philippines, Sri Lanka, Thailand (High PEP use); Asia 3 comprises Bhutan, Nepal, Bangladesh, Pakistan (Himalayan region); Asia 2 comprises Cambodia, Myanmar, Laos, Vietnam and Democratic People’s Republic of Korea; SADC comprises countries in the Southern African Development Community, Eurasia comprises Afghanistan, Kazakhstan, Kyrgyzstan, Mongolia, the Russian Federation, Turkmenistan, Tajikistan, and Uzbekistan.

Cluster	Direct Costs	Travel costs	Lost Income	Productivity losses from premature death	Dog vaccination	Dog population management	Livestock losses	Surveillance
Asia 2	75.15	15.96	20.90	252.298	2.073	0.074	24.163	0.000
Asia 3	16.67	4.42	7.08	104.774	0.564	0.214	65.706	0.042
Asia 4	41.81	2.32	33.26	45.658	11.248	0.123	1.728	0.021
China	648.27	49.25	807.32	1,642.646	4.235	0.195	40.777	0.046
India	491.23	42.60	138.03	1,646.650	9.050	0.417	62.348	0.002
Indonesia	21.18	0.95	10.03	37.123	6.384	0.811	1.717	0.000
North Africa	38.11	1.45	19.70	106.002	2.756	1.013	89.661	0.040
Congo Basin	14.47	1.28	2.33	154.424	0.481	0.003	19.670	0.001
West Africa	48.53	2.94	5.37	313.348	6.684	0.026	60.086	0.004
SADC	55.00	4.81	19.61	199.579	4.600	0.263	110.129	0.079
Andean	32.00	1.24	27.26	7.867	10.753	0.396	3.130	0.014
Brazil	45.37	1.82	63.36	11.070	16.620	0.342	0.007	0.288
Caribbean	11.66	0.24	2.43	7.702	2.575	0.113	0.296	0.006
Central America	34.13	0.49	11.74	1.873	31.308	0.809	0.001	0.020
Southern Cone	6.18	0.13	9.00	1.730	4.710	0.521	8.753	0.007
Eastern Europe	51.09	1.84	41.88	18.350	10.460	0.053	0.627	0.062
Eurasia	28.93	2.17	68.66	89.932	4.451	1.413	13.758	0.083
Middle East	42.58	0.35	26.06	35.907	0.592	0.128	9.543	0.025
**TOTAL**	1702.35	134.28	1314.01	4676.93	129.55	6.91	512.10	0.74

## References

[1] Hampson K, Coudeville L, Lembo T, Sambo M, Kieffer A, et al (2015) Estimating the Global Burden of Endemic Canine Rabies. PLoS Negl Trop Dis 9(4): e0003709 doi: 10.1371/journal.pntd.0003709 25881058 10.1371/journal.pntd.0003709 PMC4400070

