# Supplementary material for: Estimating the Global Burden of Endemic Canine Rabies
Source: PLoS Negl Trop Dis. 2015 Apr 16;9(4):e0003709. doi: 10.1371/journal.pntd.0003709 (PMC4400070; doi:10.1371/journal.pntd.0003709)
Supplement: S1 Text — (DOCX) [file pntd.0003709.s001.docx]

**Supporting Bibliography**

Data was used from the references detailed here for the final analyses. Details of specific data used are in the compressed file archive in the *Data* folder.

1. Riacon 2009 - The second international conference of the Rabies in Asia Foundation. In: Nguyen HHT, editor; 2009; Hanoi, Vietnam.

2. Abbas SS, Venkataramanan V, Pathak G, Kakkar M, Roadmap Combat Zoonoses India R (2011) Rabies control initiative in Tamil Nadu, India: a test case for the 'One Health' approach. International Health 3: 231-239.

3. Acosta-Jamett G, Cleaveland S, Cunningham AA, Bronsvoort BMd (2010) Demography of domestic dogs in rural and urban areas of the Coquimbo region of Chile and implications for disease transmission. Preventive Veterinary Medicine 94: 272-281.

4. ACPRI (2004) Assessing burden of rabies in India: WHO sponsored national multi-centric rabies survey 2003.

5. Alavi SM, Alavi L (2008) Epidemiology of animal bites and stings in Khuzestan, Iran, 1997-2006. Journal of infection and public health 1: 51-55.

6. Ali YH, Intisar KS, Wegdan HA, Ali EB (2006) Epidemiology of rabies in Sudan. Journal of Animal and Veterinary Advances 5: 266-270.

7. Alves M, de Matos MR, Reichmann MD, Dominguez MH (2005) Estimation of the dog and cat population in the State of Sao Paulo. Revista De Saude Publica 39: 891-897.

8. APPA (Accessed in June 2014) <http://www.aspca.org>.

9. Australian Companion Animal Council Inc (2006) Contribution of the Pet Care Industry to the Australian Economy, 2006

10. Aylan O, El-Sayed AFM, Farahtaj F, Janani AR, Lugach O, et al. (2011) Report of the first meeting of the Middle East and Eastern Europe rabies Expert Bureau, Istanbul, Turkey (June 8-9, 2010). Advances in Preventive Medicine 2011: 812515-Article ID 812515.

11. Azizah D, Norhafiza H, Rohaiza Y, Zainol Y, Norazian B, et al. (2005) Rabies surveillance in Malaysia; Iskandar CTNF, Hassan L, Dhaliwal GK, Yusoff R, Omar AR, editors. 58-60 p.

12. Belotto A, Leanes LF, Schneider MC, Tamayo H, Correa E (2005) Overview of rabies in the Americas. Virus Research 111: 5-12.

13. Bijari B, Sharifzade GR, Abbasi A, Salehi S (2011) Epidemiological survey of animal bites in east of Iran. Iranian Journal of Clinical Infectious Diseases 6: 90-92.

14. Bizri AR, Alawieh A, Ghosn N, Berry A, Musharrafieh U (2013) Challenges facing human rabies control: the Lebanese experience. Epidemiology and Infection.

15. Butler JRA, Bingham J (2000) Demography and dog-human relationships of the dog population in Zimbabwean communal lands. . Veterinary Record 147: 442-446.

16. Buyuk Y, Uzun I, Aybar Y, Kurnaz G, Ozaras R (2007) Rabies in Turkey: Three human cases illustrating the importance of suspecting exposure. Wilderness & Environmental Medicine 18: 214-217.

17. Chilonda P, Otte J (2006) Indicators to monitor trends in livestock production at national, regional and international levels. Livestock Research for Rural Development 18: 117.

18. Dao S, Abdillahi AM, Bougoudogo F, Toure K, Simbe C (2006) [Epidemiological aspects of human and animal rabies in the urban area of Bamako, Mali]. Bulletin de la Societe de pathologie exotique (1990) 99: 183-186.

19. David D, Dveres N, Davidson I, Yagil J, Dvorkin Z, et al. (2012) Geographic Translocation of Dog Rabies by Tourism. Israel Journal of Veterinary Medicine 67: 139-141.

20. David D, Dveres N, Yakobson BA, Davidson I (2009) Emergence of dog rabies in the Northern region of Israel. Epidemiology and Infection 137: 544-548.

21. David D, Yakobson BA (2011) REVIEW ARTICLE: DOGS SERVE AS A RESERVOIR AND TRANSMIT RABIES IN ISRAEL. IS HISTORY REPEATING ITSELF? Israel Journal of Veterinary Medicine 66: 3-8.

22. Deray RA (2010) Update on Rabies Control Initiatives in the Philippines. AREB 2010.

23. Deressa A, Ali A, Beyene M, Selassie BN, Yimer E, et al. (2010) The status of rabies in Ethiopia: A retrospective record review. Ethiopian Journal of Health Development 24: 127-132.

24. Dodet B (2006) Meeting report. Vaccine 24: 3045-3049.

25. Dodet B, The African Rabies Bureau (2009) The fight against rabies in Africa: From recognition to action. 27: 5027–5032.

26. El-Harrak M (2012) Epidemiological factors and control of rabies in North Africa; Fooks AR, Muller T, editors. 45-49 p.

27. El-Yuguda AD, Baba AA, Baba SSA (2007) Dog population structure and cases of rabies among dog bite victims in urban and rural areas of Borno State, Nigeria. Tropical Veterinarian 25: 34-40.

28. Esfandiari B, Youssefi MR, Fayaz A (2010) The prevalence of rabies and animal bites during 2004 to 2009 in North of Iran. Global Veterinaria 4: 536-538.

29. Eshetu Y, Bethelehem N, Girma T, Yared M, Yosef B, et al. (2002) Situation of rabies in Ethiopia: a retrospective study 1990-2000. Ethiopian Journal of Health Development 16: 105-112.

30. Eslamifar A, Ramezani A, Razzaghl-Abyaneh M, Fallahian V, Mashayekhi P, et al. (2008) Animal bites in Tehran, Iran. Archives of Iranian Medicine 11: 200-202.

31. Estrada R, Vos A, De Leon R, Mueller T (2001) Field trial with oral vaccination of dogs against rabies in the Philippines. Bmc Infectious Diseases 1: art. no.-23.

32. FEDIAF (2010) Facts & Figures 2010. The European Pet Food Industry.

33. Fevre EM, Kaboyo RW, Persson V, Edelsten M, Coleman PG, et al. (2005) The epidemiology of animal bite injuries in Uganda and projections of the burden of rabies. Tropical Medicine & International Health 10: 790-798.

34. Flores-Ibarra M, Estrella-Valenzuela G (2004) Canine ecology and socioeconomic factors associated with dogs unvaccinated against rabies in a Mexican city across the US-Mexico border. Preventive Veterinary Medicine 62: 79-87.

35. Frey J, Mindekem R, Kessely H, Doumagoum Moto D, Naïssengar S, et al. (2013) Survey of animal bite injuries and their management for an estimate of human rabies deaths in N'Djaména, Chad. Trop Med Int Health 18: 1555-1562.

36. Gongal G, Wright AE (2011) Human Rabies in the WHO Southeast Asia Region: Forward Steps for Elimination. Advances in preventive medicine: 383870-383870.

37. Hampson K (2009) Report from WHO consultancy on rabies control in Vietnam: 3-25 March 2009.

38. Hampson K, Dobson A, Kaare M, Dushoff J, Magoto M, et al. (2008) Rabies Exposures, Post-Exposure Prophylaxis and Deaths in a Region of Endemic Canine Rabies. Plos Neglected Tropical Diseases 2: e339.

39. Hildebrand e Grisi Filho JHd, Amaku M, Dias RA, Netto HM, Paranhos NT, et al. (2008) Use of geographic information systems in rabies vaccination campaigns. Revista De Saude Publica 42: 1005-1011.

40. Horton DL, Ismail MZ, Siryan ES, Wali ARA, Ab-dulla HE, et al. (2013) Rabies in Iraq: Trends in Human Cases 2001-2010 and Characterisation of Animal Rabies Strains from Baghdad. Plos Neglected Tropical Diseases 7: e2075.

41. Hossain M, Ahmed K, Bulbul T, Hossain S, Rahman A, et al. (2012) Human rabies in rural Bangladesh. Epidemiology and Infection 140: 1964-1971.

42. Hossain M, Bulbul T, Ahmed K, Ahmed Z, Salimuzzaman M, et al. (2011) Five-year (January 2004-December 2008) surveillance on animal bite and rabies vaccine utilization in the Infectious Disease Hospital, Dhaka, Bangladesh. Vaccine 29: 1036-1040.

43. Hu R, Tang Q, Tang J, Fooks AR (2009) Rabies in China: An Update. Vector-Borne and Zoonotic Diseases 9: 1-11.

44. Jemberu WT, Molla W, Almaw G, Alemu S (2013) Incidence of Rabies in Humans and Domestic Animals and People's Awareness in North Gondar Zone, Ethiopia. Plos Neglected Tropical Diseases 7: E2216-E2216.

45. Kamsing A, Nasipaseuth P, Archkhawong S, Southalack K, Theppangna W, et al. (2012) A review of rabies surveillance and response activities in Lao PDR to 2011. International Journal of Infectious Diseases 16: E454-E454.

46. Kato M, Yamamoto H, Inukai Y, Kira S (2003) Survey of the stray dog population and the health education program on the prevention of dog bites and dog-acquired infections: A comparative study in Nepal and Okayama prefecture, Japan. Acta Medica Okayama 57: 261-266.

47. Kayali U, Mindekem R, Hutton G, Ndoutamia AG, Zinsstag J (2006) Cost-description of a pilot parenteral vaccination campaign against rabies in dogs in N'Djamena, Chad. Tropical Medicine & International Health 11: 1058-1065.

48. Kayali U, Mindekem R, Yemadji N, Oussiguere A, Naissengar S, et al. (2003) Incidence of canine rabies in N'Djamena, Chad. Preventive Veterinary Medicine 61: 227-233.

49. Kent SJW, Naicker B, Wood DR (2012) Demographics and management of dog-bite victims at a level two hospital in KwaZulu-Natal. Samj South African Medical Journal 102: 845-847.

50. Kilic B, Unal B, Semin S, Konakci SK (2006) An important public health problem: rabies suspected bites and post-exposure prophylaxis in a health district in Turkey. International Journal of Infectious Diseases 10: 248-254.

51. Kim CH, Lee CG, Yoon HC, Nam HM, Park CK, et al. (2006) Rabies, an emerging disease in Korea. Journal of Veterinary Medicine Series B-Infectious Diseases and Veterinary Public Health 53: 111-115.

52. Kimron Vet Institute website (Accessed in June 2014) <http://agri3.huji.ac.il/~yakobson/rabheb/mapa2010.html>.

53. Kitala P, McDermott J, Kyule M, Gathuma J, Perry B, et al. (2001) Dog ecology and demography information to support the planning of rabies control in Machakos District, Kenya. Acta Tropica 78: 217-230.

54. Kitala PM, McDermott JJ, Kyule MN, Gathuma JM (2000) Community-based active surveillance for rabies in Machakos District, Kenya. Preventive Veterinary Medicine 44: 73-85.

55. Kittiphone Sea (2010) Country report on rabies control and prevention. ASEAN + 3 Meeting.

56. Knobel DL, Cleaveland S, Coleman PG, Fevre EM, Meltzer MI, et al. (2005) Re-evaluating the burden of rabies in Africa and Asia. Bulletin of the World Health Organization 83: 360-368.

57. Knobel DL, Laurenson MK, Kazwala RR, Boden LA, Cleaveland S (2008) A cross-sectional study of factors associated with dog ownership in Tanzania. Bmc Veterinary Research 4: (29 January 2008)-(2029 January 2008).

58. Koataj W, Milczak A, Koataj B, Sygit M, Sygit K (2012) The implementation of preventive vaccination of dogs and cats against rabies in rural areas. Environment and Pollution 1: 20-28.

59. Kongkaew W, Coleman P, Pfeiffer DU, Antarasena C, Thiptara A (2004) Vaccination coverage and epidemiological parameters of the owned-dog population in Thungsong District, Thailand. Preventive Veterinary Medicine 65: 105-115.

60. Kubheka V, Govender P, Margot B, Kuonza LR (2013) Dog bites and human rabies in the Uthungulu District of KwaZulu-Natal province, 2008-2010: a review of surveillance data. Southern African Journal of Epidemiology & Infection 28: 33-40.

61. Kumarapeli V, Awerbuch-Friedlander T (2009) Human rabies focusing on dog ecology-A challenge to public health in Sri Lanka. Acta Tropica 112: 33-37.

62. Lal P, Rawat A, Sagar A, Tiwari KN (2005) Prevalence of dog-bites in Delhi: knowledge and practices of residents regarding prevention and control of rabies. Health and Population - Perspectives and Issues 28: 50-57.

63. Lembo T, Hampson K, Haydon DT, Craft M, Dobson A, et al. (2008) Exploring reservoir dynamics: a case study of rabies in the Serengeti ecosystem. Journal of Applied Ecology 45: 1246-1257.

64. Ly S, Buchy P, Heng NY, Ong S, Chhor N, et al. (2009) Rabies Situation in Cambodia. Plos Neglected Tropical Diseases 3: e511.

65. Matter HC, Wandeler AI, Neuenschwander BE, Harischandra LPA, Meslin FX (2000) Study of the dog population and the rabies control activities in the Mirigama area of Sri Lanka. Acta Tropica 75: 95-108.

66. Mazigo HD, Okumu FO, Kweka EJ, Mnyone LL (2010) Retrospective analysis of suspected rabies cases reported at bugando referral hospital, mwanza, Tanzania. Journal of global infectious diseases 2: 216-220.

67. Mindekem R, Kayali U, Yemadji N, Ndoutamia AG, Zinsstag J (2005) [Impact of canine demography on rabies transmission in N'djamena, Chad]. Medecine tropicale : revue du Corps de sante colonial 65: 53-58.

68. Misriyah (2010) Rabies in Indonesia. AREB 2010.

69. Mkhize GC, Ngoepe EC, Du Plessis BJA, Reininghaus B, Sabeta CT (2010) Re-Emergence of Dog Rabies in Mpumalanga Province, South Africa. Vector-Borne and Zoonotic Diseases 10: 921-926.

70. Mueller T, Briggs D, Cliquet F (2008) WHO short term mission on "Assessment of the Rabies Situation in Tajikistan".

71. Mueller T, Briggs D, Cliquet F (2008) WHO short term mission on "Assessment of the Rabies Situation in Uzbekistan".

72. Munang'andu HM, Mweene AS, Siamudaala V, Muma JB, Matandiko W (2011) Rabies status in Zambia for the period 1985-2004. Zoonoses Public Health 58: 21-27.

73. Murray CJL, Ezzati M, Flaxman AD, Lim S, Lozano R, et al. (2012) GBD 2010: design, definitions, and metrics. The Lancet 380: 2063-2066.

74. Murray CJL, Lopez AD, Black R, Ahuja R, Ali SM, et al. (2011) Population Health Metrics Research Consortium gold standard verbal autopsy validation study: design, implementation, and development of analysis datasets. Population Health Metrics 9.

75. Murray CJL, Vos T, Lozano R, Naghavi M, Flaxman AD, et al. (2012) Disability-adjusted life years (DALYs) for 291 diseases and injuries in 21 regions, 1990-2010: a systematic analysis for the Global Burden of Disease Study 2010. Lancet 380: 2197-2223.

76. Najafi N, Ghasemian R (2009) Animal bites and rabies in northern Iran; 2001-2005. Iranian Journal of Clinical Infectious Diseases 4: 224-227.

77. Odontsetseg N, Uuganbayar D, Tserendorj S, Adiyasuren Z (2009) Animal and human rabies in Mongolia. Revue Scientifique Et Technique-Office International Des Epizooties 28: 995-1003.

78. OIE (2011) Rabies control - towards sustainable rabies prevention at the source, <http://www.oie.int/doc/ged/d12061.pdf>.

79. Pan American Health Organization/ Pan American Foot-and-Mouth Disease Center - Veterinary Public Health Unit (2010) XIII REDIPRA – Meeting of the National Directors of Rabies Control and Prevention Programs in Latin America and Caribbean Region, Buenos Aires: Argentina. (<http://new.paho.org/panaftosa/index.php?option=com_content&task=view&id=650&Itemid=336>)

80. Pfukenyi DM, Chipunga SL, Dinginya L, Matenga E (2010) A survey of pet ownership, awareness and public knowledge of pet zoonoses with particular reference to roundworms and hookworms in Harare, Zimbabwe. Tropical Animal Health and Production 42: 247-252.

81. Pfukenyi DM, Pawandiwa D, Makaya PV, Ushewokunze-Obatolu U (2007) A retrospective study of rabies in humans in Zimbabwe, between 1992 and 2003. Acta Tropica 102: 190-196.

82. Phounphenghaek K (2010) Rabies Situation in Lao PDR. AREB 2010.

83. Rabies Bulletin Europe (Accessed in June 2014) <http://www.who-rabies-bulletin.org/>.

84. RABMED control (Accessed in June 2014) <http://www.rabmedcontrol.org/>.

85. Ratsitorahina M, Rasambainarivo JH, Raharimanana S, Rakotonandrasana H, Andriamiarisoa MP, et al. (2009) Dog ecology and demography in Antananarivo, 2007. Bmc Veterinary Research 5: (01 June 2009)-(2001 June 2009).

86. Rezaeinasab M, Rad I, Bahonar AR, Rashidi H, Fayaz A, et al. (2007) The prevalence of rabies and animal bites during 1994 to 2003 in Kerman province, southeast of Iran. Iranian Journal of Veterinary Research 8: 343-350.

87. Scott-Orr H, Bingham J, Saunders G, Dibia IN, Putra AAG, et al. (2009) Potential eradication of rabies from Flores in Indonesia. 12th International Symposia on Veterinary Epidemiology and Economics (ISVEE). Durban, South Africa.

88. Shim E, Hampson K, Cleaveland S, Galvani AP (2009) Evaluating the cost-effectiveness of rabies post-exposure prophylaxis: a case study in Tanzania. Vaccine 27: 7167-7172.

89. Si H, Guo Z-M, Hao Y-T, Liu Y-G, Zhang D-M, et al. (2008) Rabies trend in China (1990-2007) and post-exposure prophylaxis in the Guangdong province. BMC Infectious Diseases 8.

90. Song M, Tang Q, Wang D-M, Mo Z-J, Guo S-H, et al. (2009) Epidemiological investigations of human rabies in China. BMC Infectious Diseases 9.

91. Sudarshan MK, Madhusudana SN, Mahendra BJ, Rao NSN, Ashwath Narayana DH, et al. (2007) Assessing the burden of human rabies in India: results of a national multi-center epidemiological survey. International Journal of Infectious Diseases 11: 29-35.

92. Sudarshan MK, Madhusudana SN, Mahendra BJ, Rao NSN, Narayana DHA, et al. (2007) Assessing the burden of human rabies in India: results of a national multi-center epidemiological survey. International Journal of Infectious Diseases 11: 29-35.

93. Sudarshan MK, Mahendra BJ, Madhusudana SN, Ashwoath Narayana DH, Rahman A, et al. (2006) An epidemiological study of animal bites in India: results of a WHO sponsored national multi-centric rabies survey. The Journal of communicable diseases 38: 32-39.

94. Sudarshan MK, Mahendra BJ, Narayan DHA (2001) A community survey of dog bites, anti-rabies treatment, rabies and dog population management in Bangalore City. Journal of Communicable Diseases 33: 245-251.

95. Suraweera W, Morris SK, Kumar R, Warrell DA, Warrell MJ, et al. (2012) Deaths from Symptomatically Identifiable Furious Rabies in India: A Nationally Representative Mortality Survey. PLoS Negl Trop Dis 6: e1847.

96. Suzuki K, Pereira JAC, Frias LA, Lopez R, Mutinelli LE, et al. (2008) Rabies-vaccination coverage and profiles of the owned-dog population in Santa Cruz de la Sierra, Bolivia. Zoonoses and Public Health 55: 177-183.

97. Tenzin, Dhand NK, Dorjee J, Ward MP (2011) Re-emergence of rabies in dogs and other domestic animals in eastern Bhutan, 2005-2007. Epidemiology and Infection 139: 220-225.

98. Tenzin, Dhand NK, Gyeltshen T, Firestone S, Zangmo C, et al. (2011) Dog Bites in Humans and Estimating Human Rabies Mortality in Rabies Endemic Areas of Bhutan. Plos Neglected Tropical Diseases 5: e1391.

99. Tenzin, Dhand NK, Rai BD, Changlo, Tenzin S, et al. (2012) Community-based study on knowledge, attitudes and perception of rabies in Gelephu, south-central Bhutan. International Health 4: 210-219.

100. Tenzin, Dhand NK, Ward MP (2012) Anthropogenic and environmental risk factors for rabies occurrence in Bhutan. Preventive Veterinary Medicine 107: 21-26.

101. Tenzin, Dhand NK, Ward MP (2011) Human rabies post exposure prophylaxis in Bhutan, 2005-2008: Trends and risk factors. Vaccine 29: 4094-4101.

102. Tenzin, Dhand NK, Ward MP (2011) Patterns of Rabies Occurrence in Bhutan between 1996 and 2009. Zoonoses and Public Health 58: 463-471.

103. Tenzin, Sharma B, Dhand NK, Timsina N, Ward MP (2010) Reemergence of Rabies in Chhukha District, Bhutan, 2008. Emerging infectious diseases 16: 1925-1930.

104. Tenzin, Wangdi K, Ward MP (2012) Human and animal rabies prevention and control cost in Bhutan, 2001-2008: The cost-benefit of dog rabies elimination. Vaccine 31: 260-270.

105. Thiptara A, Atwill ER, Kongkaew W, Chomel BB (2011) Epidemiologic Trends of Rabies in Domestic Animals in Southern Thailand, 1994-2008. American Journal of Tropical Medicine and Hygiene 85: 138-145.

106. Tiembre I, Dagnan S, Douba A, Adjogoua EV, Bourhy H, et al. (2010) Epidemiologic monitoring of human rabies in an endemic canine rabies area in the Ivory Coast. Medecine Et Maladies Infectieuses 40: 398-403.

107. Vos A, Un H, Hampson K, De Balogh K, Aylan O, et al. (2014) Bovine rabies in Turkey: patterns of infection and implications for costs and control. Epidemiology and Infection 142: 1925-1933.

108. Wang X, Ding S, Li Z, Wang L, Kou Z, et al. (2010) Human Rabies Epidemiology in Shandong Province, China. Japanese Journal of Infectious Diseases 63: 323-326.

109. Weyer J, Szmyd-Potapczuk AV, Blumberg LH, Leman PA, Markotter W, et al. (2011) Epidemiology of human rabies in South Africa, 1983-2007. Virus Research 155: 283-290.

110. Win Mei Thein (2010) Rabies Situation in Myanmar. AREB 2010.

111. Yakobson BA, King R, Sheichat N, Eventov B, David D (2008) Assessment of the efficacy of oral vaccination of livestock guardian dogs in the framework of oral rabies vaccination of wild canids in Israel. In: Dodet B, Fooks AR, Miller T, Tordo N, editors. Towards the Elimination of Rabies in Eurasia. pp. 151-156.

112. Yin C-p, Zhou H, Wu H, Tao X-y, Rayner S, et al. (2012) Analysis on factors related to rabies epidemic in China from 2007-2011. Virologica Sinica 27: 132-143.

113. Zaidi SMA, Labrique AB, Khowaja S, Lotia-Farrukh I, Irani J, et al. (2013) Geographic Variation in Access to Dog-Bite Care in Pakistan and Risk of Dog-Bite Exposure in Karachi: Prospective Surveillance Using a Low-Cost Mobile Phone System. Plos Neglected Tropical Diseases 7.
